# Climate-dependence of ecosystem services in a nature reserve in northern China

**DOI:** 10.1371/journal.pone.0192727

**Published:** 2018-02-13

**Authors:** Jiaohui Fang, Huali Song, Yiran Zhang, Yanran Li, Jian Liu

**Affiliations:** 1 Institute of Environmental Research, Shandong University, Jinan, China; 2 Wendeng Meteorological Bureau, Weihai, China; 3 Shenyang Academy of Environmental Sciences, Shenyang, China; Chinese Academy of Forestry, CHINA

## Abstract

Evaluation of ecosystem services has become a hotspot in terms of research focus, but uncertainties over appropriate methods remain. Evaluation can be based on the unit price of services (services value method) or the unit price of the area (area value method). The former takes meteorological factors into account, while the latter does not. This study uses Kunyu Mountain Nature Reserve as a study site at which to test the effects of climate on the ecosystem services. Measured data and remote sensing imagery processed in a geographic information system were combined to evaluate gas regulation and soil conservation, and the influence of meteorological factors on ecosystem services. Results were used to analyze the appropriateness of the area value method. Our results show that the value of ecosystem services is significantly affected by meteorological factors, especially precipitation. Use of the area value method (which ignores the impacts of meteorological factors) could considerably impede the accuracy of ecosystem services evaluation. Results were also compared with the valuation obtained using the modified equivalent value factor (MEVF) method, which is a modified area value method that considers changes in meteorological conditions. We found that MEVF still underestimates the value of ecosystem services, although it can reflect to some extent the annual variation in meteorological factors. Our findings contribute to increasing the accuracy of evaluation of ecosystem services.

## Introduction

Ecosystems provide a range of services to humankind that are the foundation of economic and social sustainable development [1]. The evaluation of ecosystem services is considered an effective tool for efficient allocation of limited environmental resources [2]. Although a range of methods for evaluating the value of ecosystem services exists, methods can still be controversial [3, 4]. Evaluation methods can be roughly divided into two kinds: one is based on the unit price of services (services value method) [5], the other on the unit price of the area (area value method) [6]. The services value method can simulate the value of small regional ecosystem services by establishing a production equation between a single ecosystem service and variables of the local ecological environment [7]. Nevertheless, this method requires numerous input parameters and involves a complicated calculation process [8]. The area value method is calculated by multiplying the known value of a unit of area of a particular ecosystem type by the corresponding area of that ecosystem. This is an indirect method of evaluating ecosystem services value and is suitable for large-scale assessments [9, 10].

A key difference between these two methods is that the services value method takes into account the impact of meteorological factors on ecosystem services, whereas the area value method does not [6]. Do meteorological factors have to be considered when estimating the values of ecosystem services? The effect of meteorological factors on the value of ecosystem services is still unclear. There is an adjusted area value method—the modified equivalent value factors (MEVF) method—that was modified for dynamic evaluation of Chinese terrestrial ecosystem service values [11]. The impact of meteorological factors on ecosystem service value is taken into account in this method [12]. MEVF does not require extensive data and has been widely used in evaluating the value of ecosystem services [11]. However, the accuracy of this method remains to be verified.

Most ecosystem services are positively correlated with net primary productivity (NPP): for example, raw materials and food production, gas regulation, climate regulation, environmental purification, maintenance of nutrient circulation and biodiversity, and aesthetic landscape function. Only a few ecosystem services are closely related to precipitation and other factors: for example, soil conservation, water resources supply, and hydrological regulation [12]. In this study, ecosystem services were divided into two categories: those related to NPP and those related to precipitation. Gas regulation and soil conservation were selected as representative of these two categories in order to study the impact of meteorological factors on ecosystem services.

The value of ecosystem services in a region is mainly affected by changes in land use pattern, human disturbance, and variation in annual meteorological factors [13]. Due to the strict protection of nature reserves, the influence of changes in land use pattern and human disturbance on the value of ecosystem services is less than that of other areas. Thus, the impact of meteorological factors on ecosystem service value can be more accurately analyzed in nature reserves.

Forests are one of the most important ecosystems on Earth [14]. Kunyu Mountain was selected as the study area; it is a forest nature reserve located in northern China that contains the world’s best-preserved red pine forest [15]. Measured data and remote sensing imagery coupled with a geographic information system (GIS) were used to evaluate the ecosystem service values in Kunyu Mountain Nature Reserve from 2001 to 2015. The effects of meteorological factors on ecosystem services were analyzed. The purpose of this study was to assess the rationality of each ecosystem services evaluation method, by analyzing the impact of meteorological factors on ecosystem services value. This study provides a reference for the accurate evaluation of ecosystem services.

## Methods

### Study area

**Fig 1:**
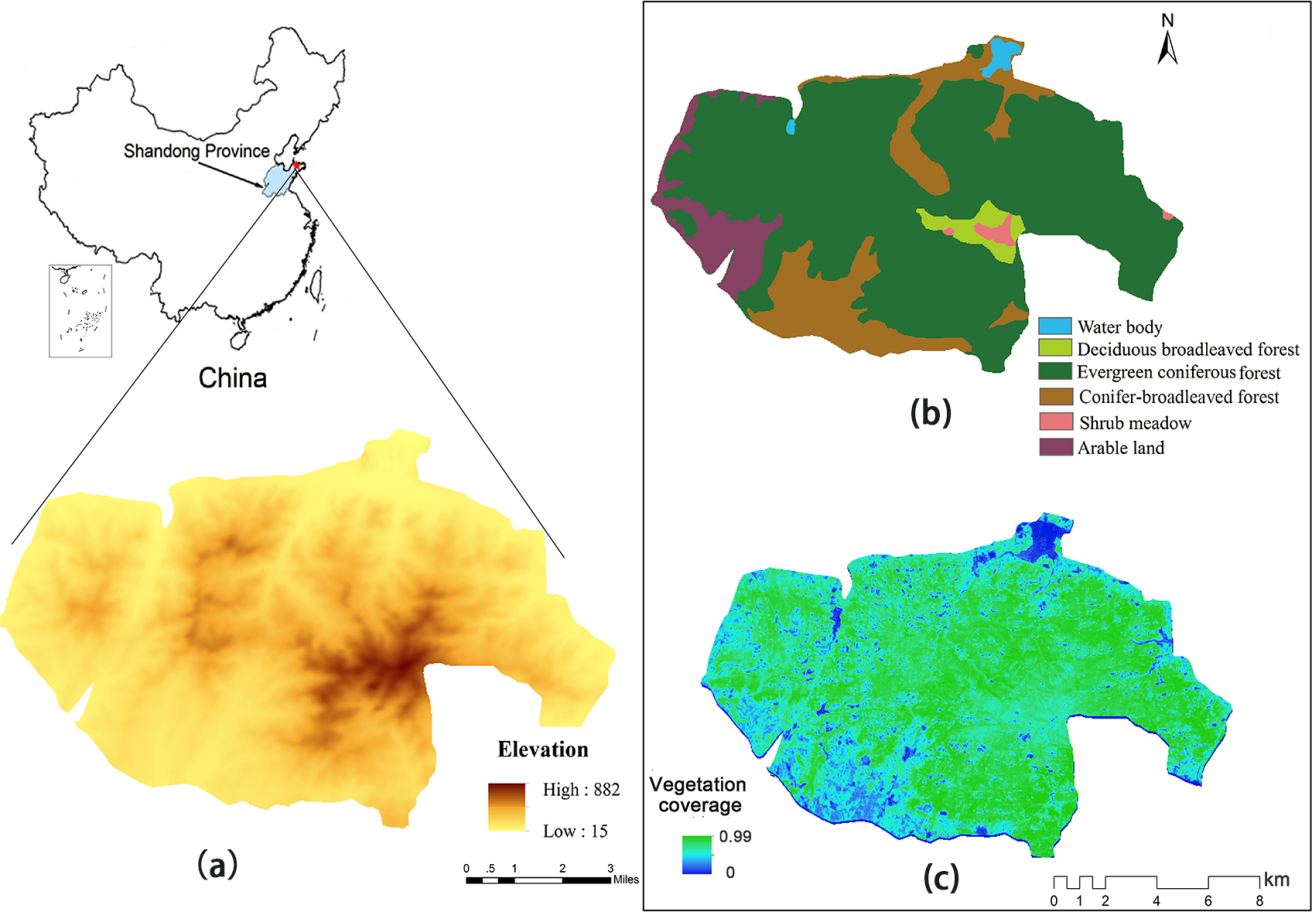
Location and vegetation cover of the Kunyu Mountain Nature Reserve, China. (a) location and topography; (b) vegetation cover type; and (c) vegetation coverage.

Kunyu Mountain Nature Reserve is located in the east of Shandong Peninsula, the largest peninsula in China, with an area of 16 076 ha (121° 37′ 0′′–121° 51′ 0′′ E, 37° 12′ 20′′–37° 18′ 50′′ N) (Fig 1A). This nature reserve is a part of the Changbai Mountains and the elevation of its main peak (Tai Bo Ding) is 923 m, with a relative elevation of nearly 900 m. The reserve has a warm temperate continental monsoon climate with four distinct seasons, humid air, and abundant light. The annual average temperature is 11.9°C, and annual rainfall is 650–900 mm. This area is acknowledged as China’s largest and the world’s best-preserved natural Japanese red pine ecosystem, which provides an excellent natural habitat for biodiversity [15].

Kunyu Mountain Nature Reserve was designated as a Provincial Nature Reserve in 2000 and a National Nature Reserve in 2007. There are five land cover types in this area: water bodies (WB), deciduous broadleaved forest (DBF), evergreen coniferous forest (ECF), conifer-broadleaved forest (CBF), shrub meadow (SM), and arable land (AL) (Fig 1B).

### Data sources

Vector data of vegetation cover types, remote sensing imagery, and meteorology in Kunyu Mountain Nature Reserve were the main inputs needed to calculate gas regulation value. Vector data of vegetation types were obtained from the Kunyu Mountain Forestry Bureau and were used to calculate the maximum light-energy utilization. Landsat TM and Landsat 8 OLI data from 2001 to 2015, at a spatial resolution of 30 m, were downloaded from USGS National Map Viewer (http://viewer.nationalmap.gov/viewer/) and used to calculate the monthly Normalized Difference Vegetation Index (NDVI). Meteorology data from 2001 to 2015, including precipitation, temperature, and solar radiation, were obtained from Meteorological Bureaus around Kunyu Mountain Nature Reserve, and these data were spatially interpolated using kriging [16].

Precipitation data, soil particle size distribution, vegetation coverage distribution, and a digital elevation model (DEM) of Kunyu Mountain Nature Reserve were the main data needed to calculate soil conservation. Precipitation data were obtained from Meteorological Bureaus around Kunyu Mountain Nature Reserve. Soil particle size distribution was measured by means of the micro-pipette method [17]. Vegetation coverage distribution was calculated using NDVI [18]. The DEM, at a spatial resolution of 30 m, was downloaded from GS Cloud (http://www.gscloud.cn/) and was used to calculate the slope length and slope steepness.

NDVI data for 2012 were not available because most remote sensing imageries in that year were obscured by clouds. Precipitation data for 2014 were missing. Thus, gas regulation and soil conservation data in 2012 and 2014 are not shown in this study.

### Estimation of gas regulation value

Oxygen release and carbon sequestration from vegetation are the main values of gas regulation. When 1 g of dry matter is produced during photosynthesis, 1.63 g carbon dioxide must be fixed and 1.19 g oxygen released. Thus, the calculation of carbon fixation and oxygen release is as follows:

$$V=S_{i}\times B_{i}\times 650\times 10^{-6}+1.19/0.44\times S_{i}\times B_{i}\times 400\times 10^{-6}$$ (1)

where *V* is the value of gas regulation (yuan); *S*_*i*_ is the area of ecosystem *i* (m^2^); and *B*_*i*_ is the NPP of ecosystem *i* (gC m^−2^ a^−1^). NPP reflects not only the productivity of a plant community in its natural environment, but also its carbon sequestration ability [19]. The unit price of carbon sequestration is 650 yuan t^−1^, which is the average Swiss carbon tax and afforestation cost price; and the unit price of oxygen release is 400 yuan t^−1^, which is the price of industrial oxygen production.

The CASA model, a remote sensing-based light use efficiency model, was used to compute per-pixel NPP at monthly intervals [20]. NPP is determined by two variables: absorbed photosynthetically active radiation (APAR) and light utilization efficiency (*ε*) [21].

### Estimation of soil conservation value

The value of the soil conservation service is composed of three parts: the value of soil fertility conservation, the value of reduction in silt deposition, and the value of reduction in land abandonment [22]. The value of soil conservation was calculated based on the market price, opportunity cost, and shadow project cost methods. The formulas for the estimation of soil conservation value are shown in Table 1.

**Table 1:** The formulas set for the assessment of the value of soil conservation.

The value of soil conservation		
Fertility conservation	$V_{1}=\sum_{i=1}^{3}A_{c}\times C_{i}\times P_{i}$	*V*_*1*_ is value of fertility conservation, yuan ha^-1^ a^-1^; *A*_*c*_ is soil conservation per unit area, t ha^-1^ a^-1^; *C*_*1*_ is the nitrogen content of soil, 0.0478%; *C*_*2*_ is the phosphorus content of soil, 0.0563%; *C*_*3*_ is the potassium content of soil, 1.8%; *P*_*1*_ is the value of nitrogen converted from urea, 6006 yuan t^-1^; *P*_*2*_ is the value of phosphorus converted from phosphate fertilizer, 4152.5 yuan t^-1^; *P*_*3*_ is the value of potassium converted from potash fertilizer, 3814 yuan t ^-1^.
Reduction of silt deposition	*V*_2_ = 24% × *A*_*c*_ × *C*/*ρ*	*V*_*2*_ is value of reduction of silt deposition, yuan ha^-1^ a^-1^; 24% is the percent of the soil erosion deposited in the reservoir; *C* is the cost of construction of reservoirs, 2.789 yuan m^-3^; *ρ* is woodland bulk density, 1.45 t m^-3^.
Reduction of land abandonment	*V*_3_ = *A*_*c*_ × *B*/(0.6 × 1000*ρ*)	*V*_*3*_ is value of reduction of land abandonment, yuan ha^-1^ a^-1^; *B* is average annual income of forestry, 282.17 yuan ha^-1^; 0.6m is thickness of ground soil.

In this study, soil conservation was computed using the USLE model coupled with GIS. USLE is a model to assess soil erosion that has been widely applied [23–25]. GIS-based data can provide more accurate information on the study area, such as land cover types and terrain. The USLE model combined with GIS can be used to analyze soil loss and soil conservation in more detail, since the relevant processes have spatially distributed characteristics. Soil conservation was estimated by the following empirical equations [26–28]:

$$A_{c}=A_{P}-A_{a}$$ (2)

$$A_{P}=R\times K\times LS$$ (3)

$$A_{a}=R\times K\times LS\times C\times P$$ (4)

where *A*_*c*_ is soil conservation per unit area (t ha^−1^ a^−1^); *A*_*p*_ and *A*_*a*_ are potential and actual soil loss (t ha^−1^ a^−1^), respectively; *R* is the rainfall erosivity factor (MJ mm ha^−1^ h^−1^ a^−1^); *K* is the soil erosion factor (t ha^−1^ h ha^−1^ MJ^−1^ mm^−1^); *LS* is the topographic factor; *C* is the vegetation cover factor; and *P* is the conservation supporting practice factor.

#### Rainfall erosivity factor (*R*)

Rainfall erosivity factor (*R*) is an indicator of the impact of rainfall on soil erosion, indicating the potential erosion ability of rainfall on soil. This factor has a direct relationship with soil loss [29]. In this study, monthly rainfall data from 2001 to 2015 were used to calculate *R* by means of the following formula [26]:

$$R=\sum_{i=1}^{12}1.735\times 10^{\left(1.5{log}_{10}(\frac{P_{i}^{2}}{P})-0.08188\right)}$$ (5)

where *R* is the rainfall erosivity factor (MJ mm hm^−2^ h^−1^ a^−1^); *P*_*i*_ is monthly rainfall (mm); and *P* is annual rainfall (mm).

#### Soil erosion factor (K)

Soil erosion factor (*K*) indicates the vulnerability of soil to rainfall erosion and runoff [30]. In this study, *K* was determined using the EPIC equation [31]:

$$\begin{array}{l} K=\\ 0.1318\times \left\{0.2+0.3exp\lceil -0.0256S_{d}(1-\frac{S_{i}}{100})\rceil \right\}\times {\left(\frac{S_{i}}{S_{e}+S_{i}}\right)}^{0.3}\times \lfloor 1.0-\frac{0.25S_{c}}{C+exp(3.72-2.59S_{c})}\rfloor \times \\ \left\lceil 1.0-\frac{0.7SNI}{SNI+exp(-5.51+22.95SNI)}\right\rceil \end{array}$$ (6)

where *S*_*d*_, *S*_*i*_, *S*_*e*_, and *S*_*c*_ are the percentage of sand, silt, clay and organic carbon, respectively (%); SNI equates to 1 − S_d_/100; and 0.1318 is the conversion factor of US customary to SI units (t ha h ha^−1^ MJ^−1^ mm^−1^).

#### Topographic factor (*LS*)

Topographic factor (*LS*) reflects the effect of slope length and gradient on soil loss [32]. Length factor (*L*) indicates the proportion of soil loss in the field to the corresponding soil loss in an area of 22.13 m slope length. The formula is expressed as follows [33]:

$$L={\left(\frac{\lambda }{22.13}\right)}^{m}\;\left\{ \begin{array}{l} m=0.5\;\theta \geq 9\\ m=0.4\;9>\theta \geq 3\\ m=0.3\;3>\theta \geq 1\\ m=0.2\;1\geq \theta \end{array}\right.$$ (7)

where λ is slope length (m); *m* is the slope length coefficient; and *θ* is the gradient.

The slope steepness factor (*S*) is expressed as follows [34]:

$$S=\left\{ \begin{array}{l} 10.8sin\theta +0.03,\;\theta <9\%\\ 16.8sin\theta -0.05,\;9\%\leq \theta \leq 18\%\\ 21.91sin\theta -0.96,\;\theta >18\% \end{array}\right.$$ (8)

#### Vegetation cover factor (*C*)

There is negative correlation between soil loss and vegetation coverage, and the turning point is at about 78.3% [35]. The vegetation cover factor (*C*) is expressed as follows:

$$C=\left\{ \begin{array}{l} 1\;ƒ=0\\ 0.6508-0.3436lgƒ\;0<ƒ\leq 78.3\%\\ 0\;ƒ>78.3\% \end{array}\right.$$ (9)

where *f* is vegetation coverage (%).

#### Conservation supporting practice factor (*P*)

Conservation supporting practice factor (*P*) is the ratio of soil loss with specific supporting practices to soil loss with up and down slope cultivation. Specific support practices include contouring, strip cropping, terracing, and subsurface drainage. The value of *P* ranges from 0.1 to 1; values defined for each land use type are shown in Table 2 [36].

**Table 2:** P value in the study area.

Land cover type	P factor
Forest land	0.1
Built-up land	1
Arable land	0.4
Water body	0.5
Waste land	1

### Correlation analysis

Two methods were used to analyze the correlation between meteorological factors and ecosystem services. The first method, Pearson correlation, analyzes the correlation between the average ecosystem services value of each land cover type and the corresponding average of meteorological data in the study area. The second method, spatial correlation analysis, uses the correlation coefficient to analyze the correlation between ecosystem services value and meteorological data in each unit grid. The spatial correlation coefficient was calculated using the following equation:

$$R_{xy}=\frac{\sum_{k=1}^{n}\left(x_{k}-\bar{x})(y_{k}-\bar{y}\right)}{\sqrt{\sum_{k=1}^{n}\left(x_{k}-\bar{x}\right)}\sqrt{\sum_{k=1}^{n}\left(y_{k}-\bar{y}\right)}}$$ (10)

where *x*_*k*_ is gas regulation, soil loss, or soil conservation in year *k*; $\bar{x}$ is the average gas regulation, soil erosion, or soil conservation for all years; *y*_*k*_ is precipitation, solar radiation or temperature in year *k*; $\bar{y}$ is the average precipitation, solar radiation or temperature for all years; and *R*_*xy*_ is the correlation coefficient of variables *x* and *y*.

The first method can quantitatively reflect correlation between ecosystem services value and meteorological factors in different types of land cover. The second method can visualize the correlation of ecosystem services value and meteorological factors, is an effective tool to respond to the spatial relations of different variables, and is used to analyze the causes of correlation distribution.

### Modified equivalent value factors

The MEVF method was proposed by Xie et al. who constructed a value table of ecosystem services equivalent per unit area (Table 3) [12]. The economic value of a standard ecosystem services equivalent is 3406.5 yuan ha^-1^ in 2010. Changes in ecosystem service value due to spatio-temporal differences, including variation in meteorological factors, are adjusted by temporal and spatial adjustment factors:

$$F_{nij}=\left\{ \begin{array}{c} P_{ij}\times F_{n1}\\ S_{ij}\times F_{n2} \end{array}\right.$$ (11)

where *F*_*nij*_ is the modified equivalent factor per unit area of ecosystem *i* in *j* months; *P*_*ij*_ is NPP spatio-temporal adjustment factor; *S*_*ij*_ is soil conservation spatio-temporal adjustment factor; *Fn*_*1*_ represents the equivalent factor of the ecosystem services positively related to NPP (e.g., raw materials and food production, gas regulation, climate regulation, environmental purification, maintenance of nutrient circulation, biodiversity, and landscape function); and *Fn*_*2*_ represents the equivalent factor of soil conservation.

**Table 3:** The value of gas regulation and soil conservation of unit area of different ecosystem types based on the MEVF method (yuan ha^-1^ a^-1^).

	Gas regulation	Soil conservation
Water body	2632.01	3168.05
Deciduous broadleaved forest	7392.11	9027.23
Evergreen coniferous forest	5791.05	7017.39
Conifer-broadleaf forest	8005.28	9742.59
Shrub meadow	6710.81	8175.6
Arable land	2282.36	3508.7

The expression of *P*_*ij*_ and *S*_*ij*_ is follows:

$$P_{ij}=B_{ij}/\bar{B}$$ (12)

$$S_{ij}=E_{ij}/\bar{E}$$ (13)

where *B*_*ij*_ is the NPP of ecosystem *i* in *j* months; $\bar{B}$ represents average annual NPP of ecosystem *i* in China; *E*_*ij*_ is the soil conservation of ecosystem *i* in *j* months; and $\bar{E}$ represents average annual soil conservation of ecosystem *i* in China.

## Results

### Annual variation in meteorological factors

**Fig 2:**
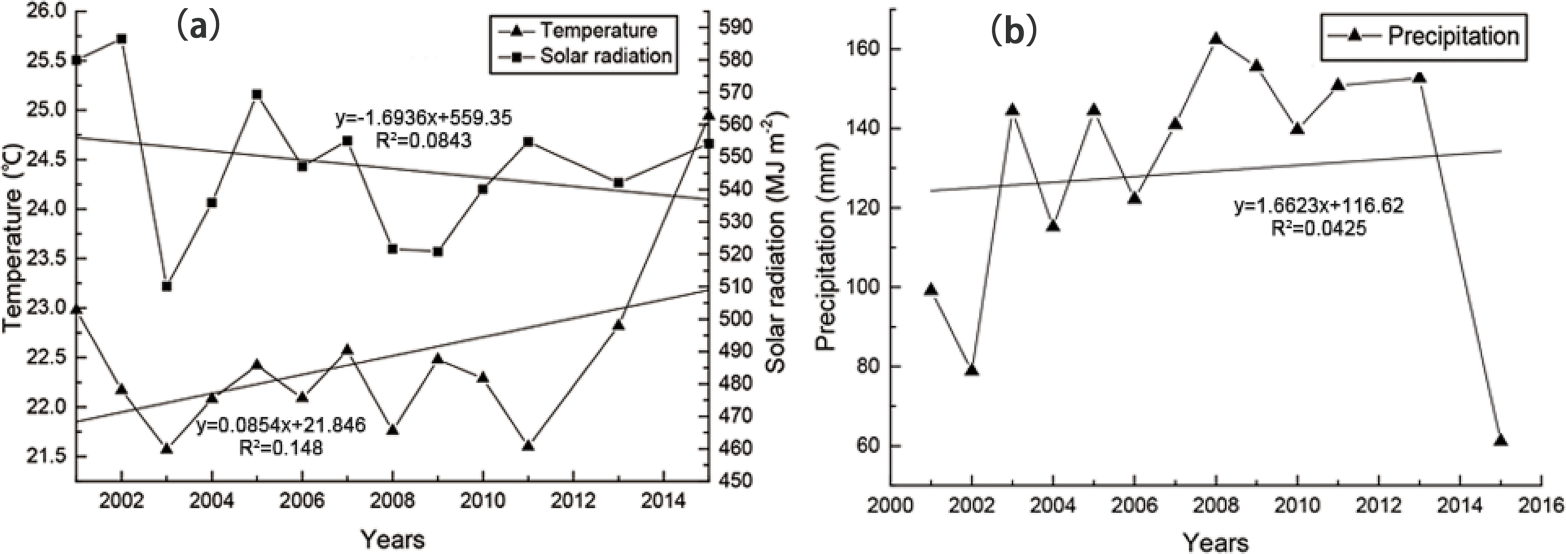
Variation in average meteorological factors from 2001 to 2015: (a) annual temperature and solar radiation; and (b) annual precipitation.

Both gas regulation and soil conservation were mainly concentrated in the period from May to September in a year in our data. Thus, average meteorological data from May to September were selected for analysis in this study. There was no significant linear trend of changes in average precipitation, temperature, and solar radiation from 2001 to 2015 (Fig 2), which illustrated that the variance laws of meteorological factors were not obvious.

### Spatial distribution of NPP and soil conservation

**Fig 3:**
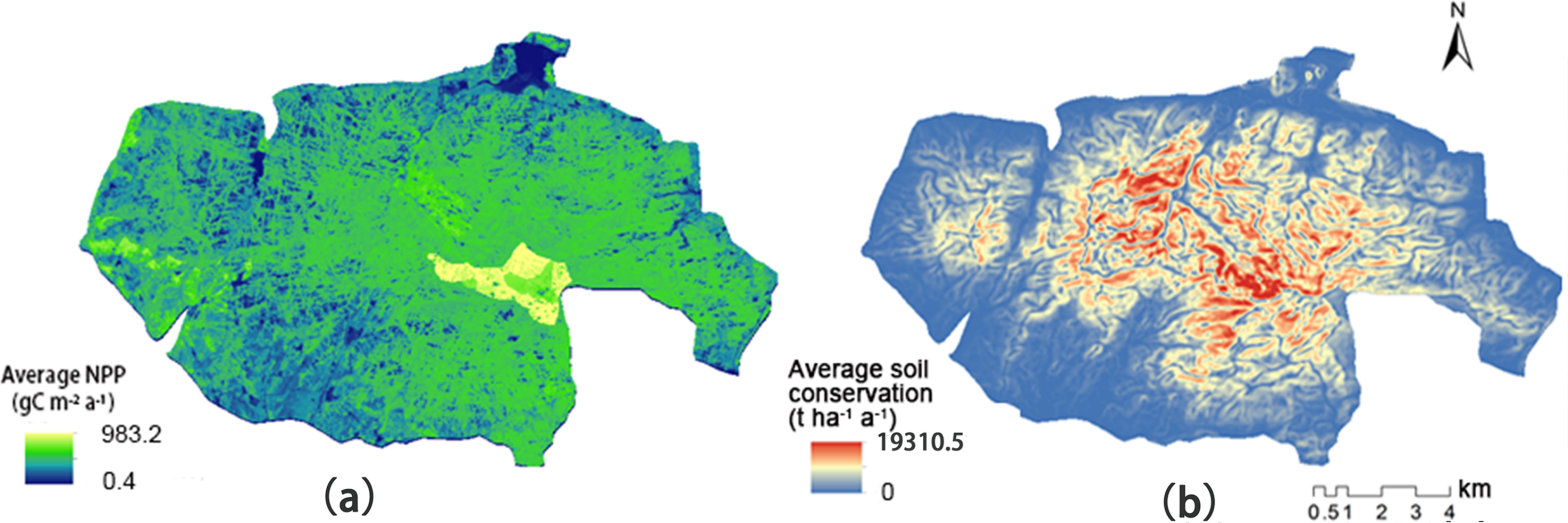
Spatial distribution of average net primary productivity (NPP) and soil conservation from 2001 to 2015: (a) NPP; and (b) soil conservation.

The spatial distribution of average NPP and soil conservation in the study area, from 2001 to 2015, was illustrated in Fig 3. The distribution of NPP gradually decreased from the center to the surrounding areas. Peak NPP appeared in the core zone of the nature reserve. The distribution of NPP was strongly correlated with the distribution of vegetation and land cover types. The lowest value of NPP was in WB (201.28 gC m^−2^ yr^−1^), followed by CBF (356.19 gC m^−2^ yr^−1^). The highest value was mainly distributed in DBF (794.74 gC m^−2^ yr^−1^). ECF made the greatest contribution to NPP, accounting for about 70%. High values for soil conservation were concentrated in mountainous areas with high altitude. The highest values of soil conservation were mainly distributed in DBF and SM, 8463.06 and 6036.44 t ha^−1^ a^−1^ respectively. ECF made the greatest contribution to soil conservation, accounting for about 80%.

### Correlation of gas regulation with meteorological factors

**Fig 4:**
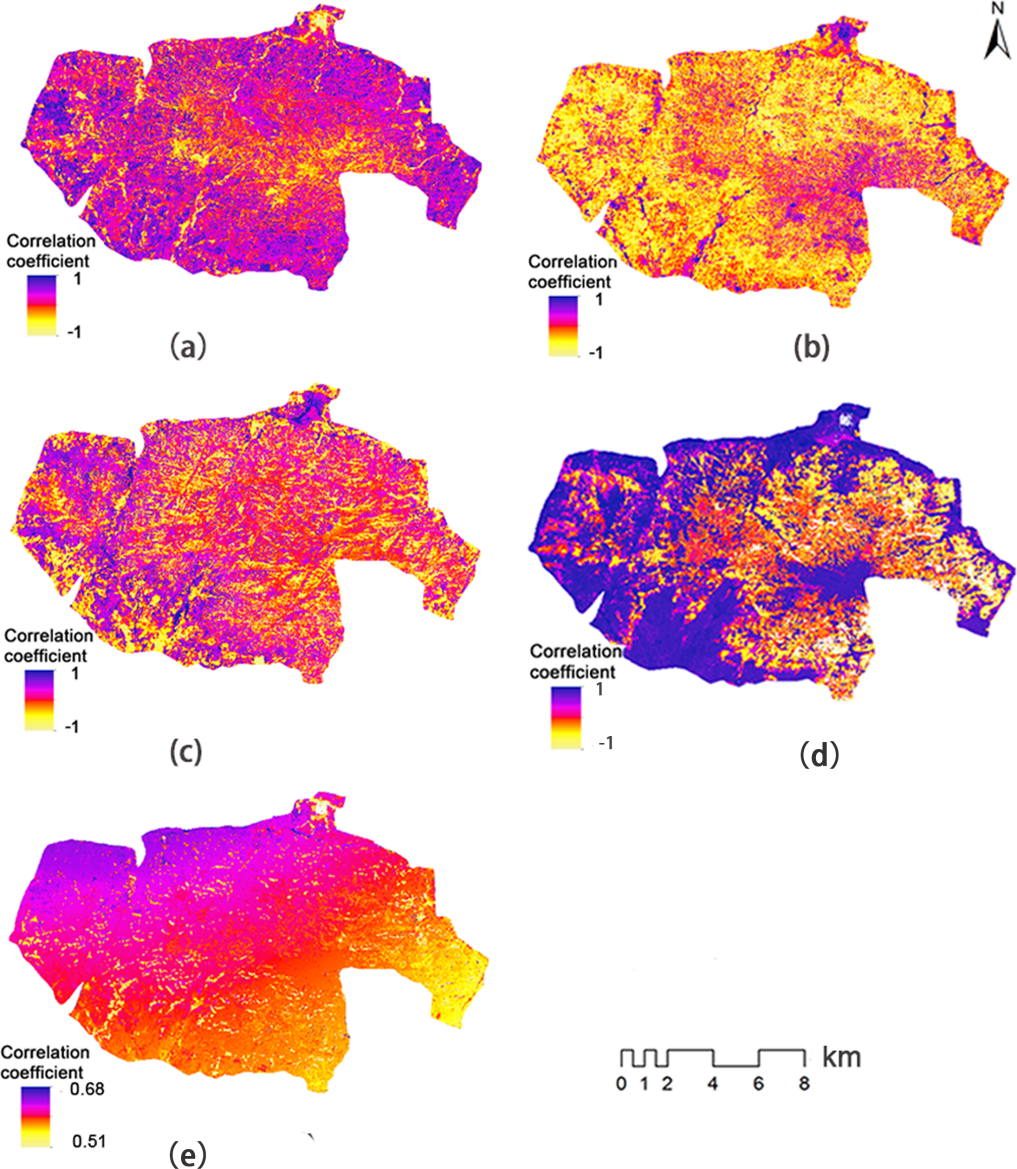
Correlation coefficient between meteorological factors and ecosystem services: (a) precipitation and gas regulation; (b) solar radiation and gas regulation; (c) temperature and gas regulation; (d) precipitation and soil loss; and (e) precipitation and soil conservation.

**Fig 5:**
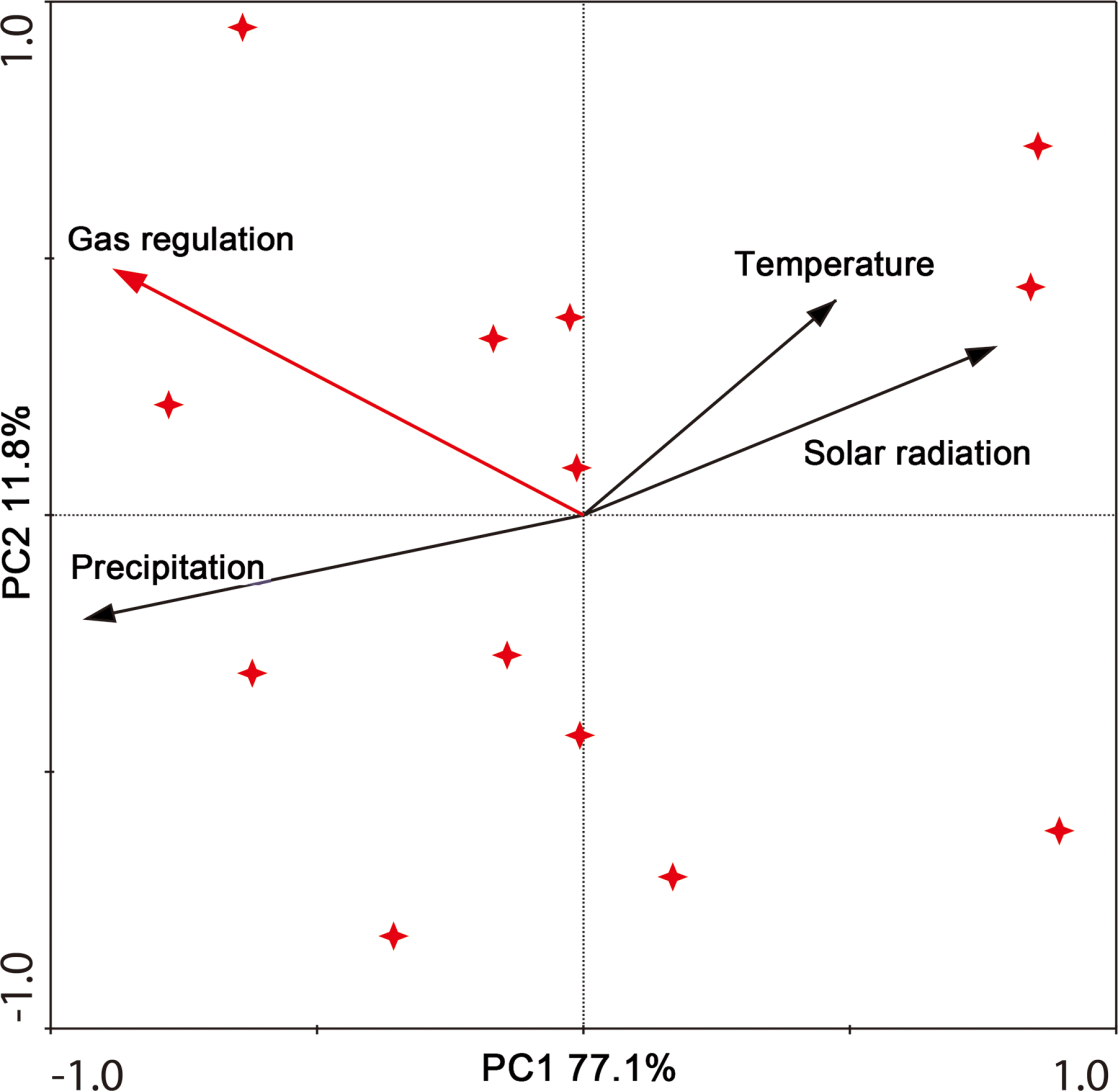
Principal component analysis (PCA).

Temperature, precipitation, and solar radiation were selected as meteorological factors that might have an impact on gas regulation. Pearson and spatial correlation analyses were used to obtain the impact of meteorological factors on gas regulation (Table 4 and Fig 4A, 4B and 4C). Through Pearson correlation analysis (Table 4), we found that precipitation was significantly positively correlated with gas regulation (P < 0.01), while solar radiation was significantly negatively correlated with gas regulation (P < 0.05). Temperature had no significant effect on gas regulation. There was a significant positive correlation between precipitation and gas regulation in half of the land cover types (ECF, CBF, and AL). However, gas regulation in WB, DBF, and SM had no significant correlation with any meteorological factor (Table 4). Through spatial correlation analysis we found that, except for the water bodies and the central part of the research area, precipitation had a significant positive correlation (P < 0.05) with gas regulation in most areas (Fig 4A) and apart from the water body and the central part of the research area, solar radiation in most areas was significantly negatively correlated (P < 0.05) with NPP. The correlation coefficient between temperature and NPP (Fig 4C) was not uniform. Most regions had weak negative correlation. By using principal component analysis (PCA) we obtained that the main meteorological factor affecting gas regulation was precipitation, followed by solar radiation (Fig 5) and there existed correlate relations among meteorological factors. Precipitation showed negative correlations with precipitation and temperature (Fig 5).

**Table 4:** Pearson correlation analysis among the value of gas regulation and meteorological factors.

	WB	ECF	DBF	CBF	SM	AL	ENR^a^	Precipitation	Temperatures	SR^b^
WB	1									
ECF	0.407	1								
DBF	0.268	0.814**	1							
CBF	0.436	0.921**	0.563*	1						
SM	0.162	0.659*	0.954**	0.378	1					
AL	0.402	0.864**	0.504	0.977**	0.327	1				
ENR^a^	0.42	0.997**	0.784**	0.946**	0.624*	0.898**	1			
Precipitation	0.405	0.675*	0.389	0.730**	0.216	0.719**	0.693**	1		
Temperatures	-0.118	-0.186	-0.181	-0.143	-0.133	-0.143	-0.182	-0.632*	1	
SR^b^	-0.402	-0.565*	-0.301	-0.561*	-0.09	-0.586*	-0.569*	-0.620*	0.299	1

^a^ represented the value of the entire nature reserve.

^b^ represented the solar radiation.

** represented correlation is significant at the 0.01 level.

* represented correlation is significant at the 0.05 level.

The above analysis showed that precipitation and solar radiation were significantly related to gas regulation in the study area. In order to obtain the contribution of these two meteorological factors to the variation in gas regulation, redundancy analysis (RDA) was carried out. The results showed that precipitation and solar radiation explained 49.3% of the total variation in the value of gas regulation. Out of this variation, precipitation contributed 99.6% and solar radiation contributed 39.6%. Thus, meteorological factors played an important role in variation in gas regulation.

**Fig 6:**
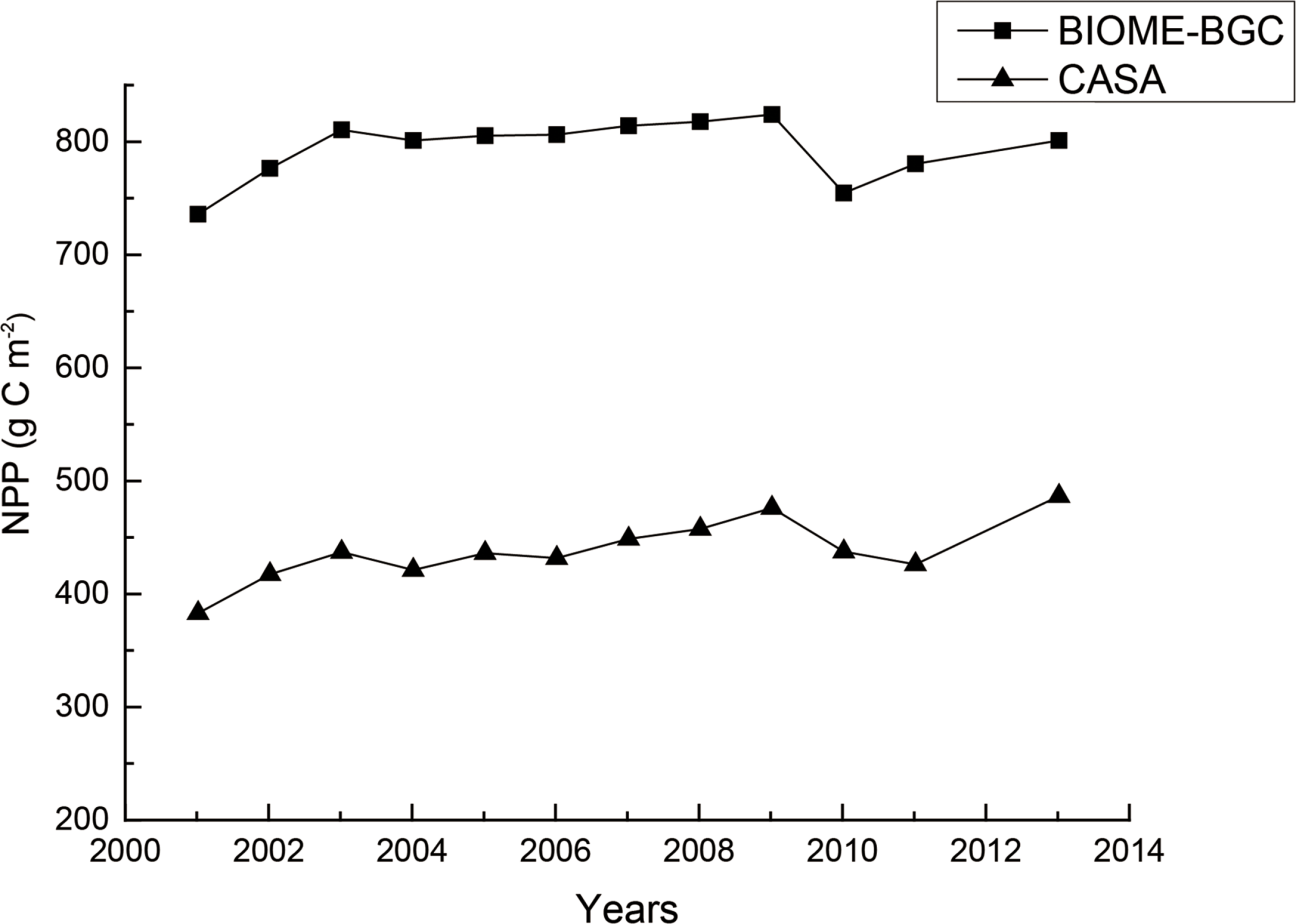
Comparison of net primary productivity (NPP) computed by the CASA and BIOME-BGC models from 2001 to 2013.

NPP calculated in this study was compared with NPP from MOD17A3 data provided by the U.S. National Aeronautics and Space Administration (NASA) from 2001 to 2013. CASA and BIOME-BGC models were used in this study and in MOD17A3 data, respectively. Comparing results of the two different models found that annual variation trends between the two results were almost identical (Fig 6). This indicated that the influence of meteorological factors on gas regulation is not caused by calculation error in the CASA model; the influence of meteorological factors on variation in gas regulation is a real phenomenon. The overall difference between the two models was large because the calculation formulas between the two models are different, and the resolution of remote sensing imagery (1 km) used by the BIOME-BGC model of MOD17A3 data was much lower than that (30 m) used by the CASA model in this study. This resulted in inaccurate values of MOD17A3 for the small study area.

### Correlation of soil conservation with meteorological factors

Under the strict protection of the natural reserve, changes in soil particle distribution and terrain topography from 2001 to 2015 were small, while precipitation and vegetation cover varied annually. Pearson and spatial correlation analyses were used to obtain the correlation between actual soil loss and precipitation (Table 5 and Fig 4D). Through Pearson correlation analysis (Table 5), we found a significant positive correlation (P < 0.05) between precipitation and soil loss in most land cover types, except ECF. The results of spatial correlation analysis (Fig 4D) were consistent with this. However, there was a negative correlation between precipitation and soil loss in part of the ECF with dense vegetation.

**Table 5:** Pearson correlation analysis among the value of soil loss and precipitation factors.

	ECF	DBF	CBF	SM	AL	ENR^a^	Precipitation
ECF	1						
DBF	0.927**	1					
CBF	0.951**	0.985**	1				
SM	0.863**	0.985**	0.969**	1			
AL	0.932**	0.977**	0.994**	0.969**	1		
ENR^a^	0.997**	0.939**	0.966**	0.883**	0.947**	1	
Precipitation	0.553	0.684**	0.641*	0.699**	0.659*	0.570*	1

^a^ represented the value of the entire nature reserve.

** represented correlation is significant at the 0.01 level.

* represented correlation is significant at the 0.05 level.

The correlation between soil conservation and average precipitation from May to September was shown in Table 6 and Fig 4E. Pearson correlation analysis (Table 6) showed that precipitation had significant positive correlation with soil conservation (P < 0.01). The results of spatial correlation analysis (Fig 4E) were consistent with this. The correlation coefficient was evenly distributed across most of the research area, ranging from 0.51 to 0.68. This means an increase in precipitation promotes growth in soil conservation value.

**Table 6:** Pearson correlation analysis among the value of soil conservation and precipitation factors.

	ECF	DBF	CBF	SM	AL	ENR^a^	Precipitation
ECF	1						
DBF	0.998**	1					
CBF	1.000**	0.998**	1				
SM	0.928**	0.929**	0.927**	1			
AL	0.994**	0.988**	0.995**	0.914**	1		
ENR^a^	1.000**	0.998**	1.000**	0.928**	0.994**	1	
Precipitation	0.712**	0.711**	0.711**	0.732**	0.718**	0.712**	1

^a^ represented the value of the entire nature reserve.

** represented correlation is significant at the 0.01 level.

### Comparison between modified equivalent value factor method and services value method

**Fig 7:**
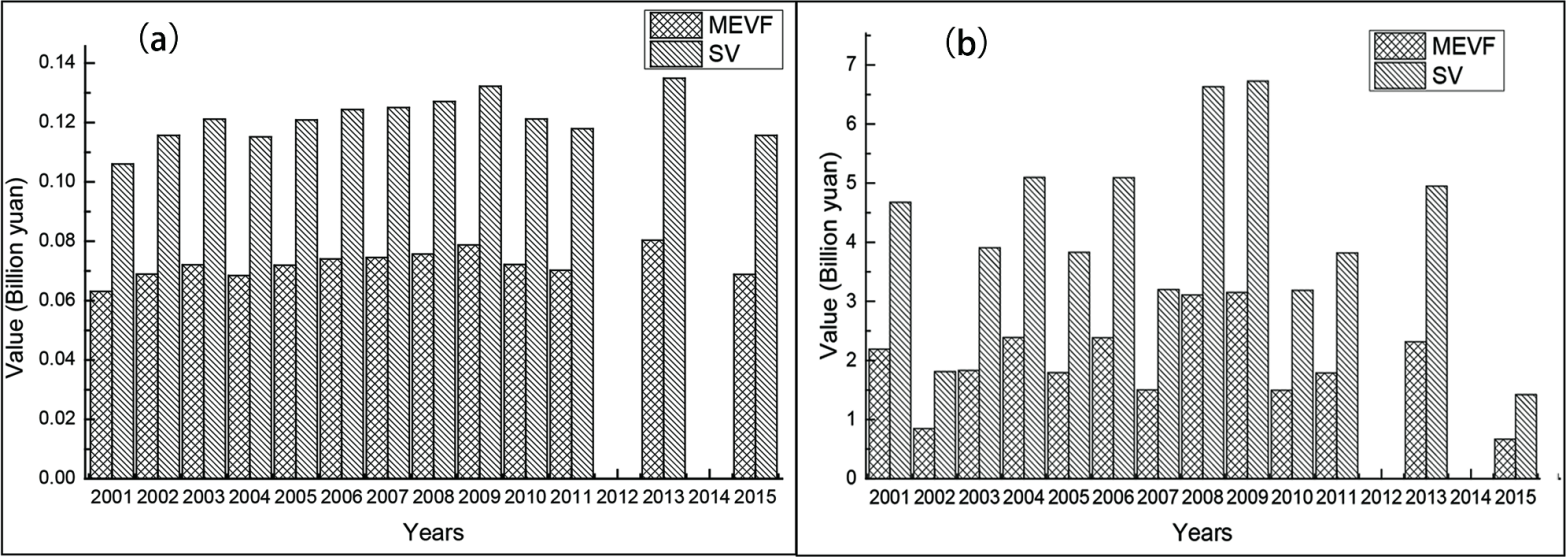
Comparison of the values of gas regulation and soil conservation between the modified equivalent value factor (MEVF) method and services value method from 2001 to 2015: (a) gas regulation; and (b) soil conservation.

To verify the accuracy of MEVF in evaluating the value of ecosystem services, we compared the value of gas regulation and soil conservation between the MEVF and services value methods from 2001 to 2015. The results suggested that the values of gas regulation and soil conservation of the services value method were two times as high as those of the MEVF method (Fig 7).

## Discussion

Our study found that precipitation had a significant positive correlation with gas regulation in most areas, which is the same as the results of Zhang et al. [37]. This is because vegetation may be subjected to water stress caused by water shortage in the study area. Photosynthesis decreases due to stomatal closure when hydraulic capacity cannot meet transpirational demand, which leads to a decrease in NPP [38, 39]. Increases in precipitation can alleviate water stress, promoting growth in NPP and gas regulation. However, there was no significant correlation between precipitation and gas regulation in the central part of the study area and the water body. According to elevation (Fig 1A), spatial correlation distribution (Fig 4A), and annual average vegetation coverage (Fig 1C), we found weak correlation in the central area mainly distributed along areas of high altitude. Vegetation coverage in the high altitude area was slightly lower than that in lower altitudes around the central area. Thus, correlation analysis in the central area might be affected by the large number of rocks distributed in high altitude mountains, resulting in a low correlation coefficient. Though the vegetation coverage in AL and CBF was also small (Fig 1C), the correlation between precipitation and gas regulation was strong. As there is a lot of arable land in these two types of areas, vegetation coverage is not as high as that in areas of trees and shrubs. Nonetheless, growth of crops shows strong sensitivity to precipitation [40], which resulted in strong correlation between precipitation and gas regulation in AL and CBF.

By comparing the spatial correlation of precipitation, solar radiation, and temperature with gas regulation (Fig 4A, 4B and 4C), it was found that distribution of the correlation coefficient of precipitation with gas regulation was the opposite of that of solar radiation with gas regulation in most areas. It was also the inverse of that of temperature with gas regulation in part of the study area. PCA showed that precipitation was negatively correlated with solar radiation and temperature. The spatial response of NPP to temperature is remarkably different to that of the response to precipitation in Heilongjiang [40]. In addition, in terms of the variation in gas regulation caused by meteorological factors, the contribution of solar radiation (39.6%) was much lower than that of precipitation (99.6%). Due to weak correlation between temperature and gas regulation, the contribution of temperature to gas regulation is small. Thus, the main meteorological factor affecting variation of gas regulation directly was precipitation and the correlation between solar radiation and gas regulation is due to the correlation between solar radiation and precipitation, although visible light from solar radiation is the raw material for producing plant organic matter through photosynthesis [41] and the impact of temperature on vegetation growth is significant. The results of Zhang et al. also shows that the effect of temperature on NPP depends heavily on precipitation [37].

Soil loss is closely related to precipitation, terrain gradient, soil properties, and vegetation coverage [18]. Among these factors, terrain gradient and soil properties are relatively stable and almost unchanged. Soil loss is mainly affected by precipitation and vegetation cover. Precipitation is the main factor causing soil erosion [18], while precipitation also affects the water content of soil, thereby affecting growth of vegetation. This study shows that increases in precipitation can promote growth of vegetation. Increases in vegetation effectively reduce soil erosion [42]. Therefore, the relationship between rainfall and soil loss is not clear because of interactions between rainfall, vegetation, and soil loss. In this study, the effect of precipitation on soil loss can be demonstrated by analysis of their spatial correlation (Fig 4D). The correlation between precipitation and soil erosion was positive in most parts of the study area; negative correlation was found only in a small number of areas with large vegetation coverage. This indicates that when precipitation increases, the positive effect of increasing vegetation on soil loss is much smaller than the negative effect of precipitation erosion on soil loss in most areas. The area of significant vegetation cover to soil loss correlation is much smaller than that of precipitation in the Loess Plateau [18]. The positive effect of vegetation cover on soil loss is greater only in areas with dense vegetation coverage. Thus, positive or negative effects of rainfall on soil loss are mainly dependent on vegetation coverage [43], suggesting the importance of protecting vegetation for soil conservation.

**Fig 8:**
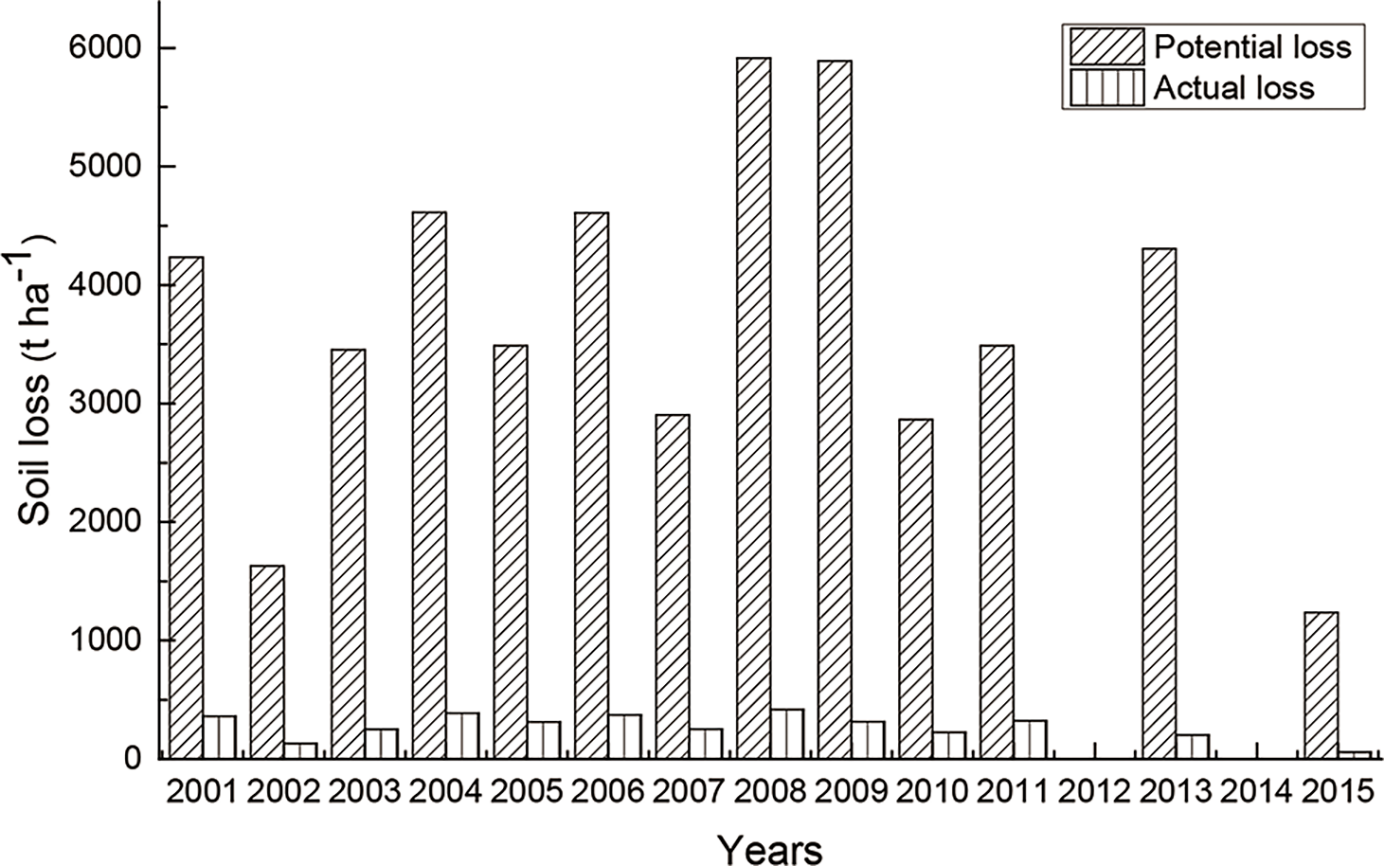
Comparison of potential and actual soil loss from 2001 to 2015.

The spatial correlation between soil conservation and precipitation was significant and positive in most of the study area. When precipitation increases, potential and actual soil losses will both increase, while actual soil erosion will be much less than potential losses because of the protection provided by vegetation cover [44] (Fig 8), resulting in increased soil conservation. In addition, the increase in precipitation promotes increases in vegetation density, reducing actual soil loss and increasing soil conservation.

Meteorological factors, especially precipitation, have a great influence on the value of gas regulation (ecosystem services related to NPP) and of soil conservation (ecosystem services related to precipitation). Therefore, when estimating ecosystem services value, it is necessary to consider variation in annual meteorological factors and climate differences of different regions. Runting et al. proposed that ignoring the impact of climate change can produce misleading assessments of ecosystem services [45]. In general area value methods, the value of unit area of different ecosystem types is not adjusted according to annual variation in meteorological factors and climate differences in different regions [9, 46, 47], which may impair the accuracy of the value of ecosystem services. MEVF, a kind of area value method, can evaluate ecosystem services considering changes in meteorological conditions in different regions or different years. However, the value of unit area obtained for different ecosystem types is not reasonable to some extent. MEVF might underestimate the value of ecosystem services in forest nature reserves, and thus cannot reflect the true ecosystem services value. The services value method, considering climate change, can lead to substantial improvement in the accuracy of the valuation of ecosystem services, although it is likely to be costly and time-consuming compared with other methods. [45].
